# Nosocomial Endocarditis Caused by *Corynebacterium amycolatum* and Other Nondiphtheriae Corynebacteria

**DOI:** 10.3201/eid0801.010151

**Published:** 2002-01

**Authors:** Karen L. Knox, Alison H. Holmes

**Affiliations:** * St. George’s Hospital, London, United Kingdom; † Imperial College School of Medicine, Hammersmith Hospital, London, United Kingdom

**Keywords:** Corynebacteria, *C. amycolatum*, nosocomial endocarditis, intravascular device

## Abstract

The nondiphtheriae corynebacteria are uncommon but increasingly recognized as important agents of community-acquired endocarditis in patients with underlying structural heart disease, as well as of prosthetic-valve endocarditis. We describe three cases of nondiphtheriae corynebacterial endocarditis, including the first reported case of endocarditis caused by *Corynebacterium amycolatum*, occurring over an 18-month period, all in association with indwelling intravascular devices.

The nondiphtheriae corynebacteria, major components of the normal flora of human skin and mucous membranes, are commonly isolated from clinical specimens. As such, they are frequently dismissed as contaminants. Recently, however, the microbiologic classification of this group of organisms and their role in clinical disease are being more clearly defined (*1*). In particular, *Corynebacterium amycolatum*, which was first described in 1988 (*2*), is becoming widely recognized as an important pathogen, although it has been underreported in part because of its misidentification as *C. xerosis* (*3*,*4*), an established human pathogen.

These organisms are causes of community-acquired endocarditis in patients who have underlying structural heart disease or are immunocompromised, as well as of prosthetic-valve endocarditis. Cases in previously healthy patients are rarely described (*5*,*6*). Hospital-acquired bacterial endocarditis accounts for 7.5% to 29% of cases of endocarditis in tertiary-care hospitals and is strongly associated with infection of indwelling intravascular lines (*7*). Common causative organisms include *Staphylococcus aureus*, enterococci, and coagulase–negative staphylococci. *Corynebacterium* species are rarely reported as agents of hospital-acquired endocarditis.

We describe three cases of endocarditis caused by three different species of corynebacteria in our hospital within an 18-month period, all associated with indwelling intravascular devices (IID). These accounted for 3 of 10 cases of hospital-acquired endocarditis identified during this period and include the first reported case of endocarditis caused by *C. amycolatum*. Two of the *Corynebacterium* species were resistant to multiple antibiotics. These cases highlight the importance of the nondiphtheriae corynebacteria as emerging multiresistant nosocomial pathogens in the growing population of patients with IID.

## Case Reports

### Case 1

A 74-year-old woman was hospitalized with a diagnosis of antineutrophil cytoplasmic antibody (ANCA)-positive vasculitis. Her treatment included daily plasma exchanges via a right internal jugular vascular catheter that was placed on the 5th hospital day. On day 21 of admission, she had a low-grade fever that resolved after removal of the catheter, the tip of which produced a culture of diphtheroids. Blood cultures were sterile.

The patient was discharged after 1 month but was readmitted 4 weeks later for control of vasculitis. On admission she was anemic (hemoglobin 7.0 g/dL) and had persistent microscopic hematuria and elevated C-reactive protein. She had no new vasculitic manifestations and, as previously, a grade II systolic murmur was noted at the left sternal edge. She had a fever of 38°C 48 hours after admission. Four sets of blood cultures were positive for *C. amycolatum* (Table 1). Transesophageal echocardiogram (TOE) showed a large mobile vegetation on the mitral valve, with prolapse and mild to moderate mitral regurgitation. A transthoracic echocardiogram on her previous admission had shown no mitral valve abnormality. A combination of intravenous vancomycin and oral rifampicin was begun, and the fever resolved within 48 hours. The patient refused surgical intervention, and dual antibiotic therapy was continued indefinitely. A follow-up TOE at 15 months showed improvement in mitral valve features and no vegetations. The antibiotics were stopped after 16 months, and 5 months later the patient had no clinical evidence of disease recurrence.

**Table 1:** Patient characteristics, heart valve affected, source of *Corynebacterium* infection and organism cultured, London

Case	Age(y)/sex	Underlying disease process	Site of endocarditis	Associated IID^a^	Therapy	Outcome
1	74/F	ANCA + vasculitis	Native mitral valve	Vascular catheter for HD	Vancomycin i.v. + oral rifampicin, 16 months^b^	Resolved at 21 months postdiagnosis
2	69/F	ANCA + vasculitis	Native mitral valve	Gortex AV fistula, vascular catheter for HD	Vancomycin i.v. + oral rifampicin, 37 days; mitral valve replacement	Died of unrelated causes 9 weeks after diagnosis
3	53/M	Postoperative acute renal failure	Prosthetic mitral valve (Starr Edwards)	Vascular catheter for HD CVC	Vancomycin i.v. + oral rifampicin, 42 days	Died of unrelated causes 8.5 months postdiagnosis

^a^Patient refused surgery and follow-up echocardiograms.

^b^IID = indwelling intravascular device; ANCA = anti-neutrophil cytoplasmic antibody; HD = hemodialysis; AV = arteriovenous; CVC = central venous catheter.

### Case 2

A 69-year-old woman with hemodialysis-dependent chronic renal failure secondary to ANCA-positive vasculitis was admitted after acute thrombosis of a GoreTex dialysis fistula in her left arm. She had required temporary vascular access for hemodialysis for the preceding 12 days. On examination she was unwell and feverish. She had a grade I systolic murmur over the cardiac apex; no previous echocardiogram was available. Two sets of blood cultures grew *C. striatum* (Table 1), as did the tip of her vascular catheter on removal. A TOE revealed a large vegetation on her mitral valve. Because of penicillin allergy, intravenous vancomycin and oral rifampicin were begun. The prosthetic arteriovenous fistula could not be removed completely, although it was considered a potential reservoir of infection. The patient had a mitral valve replacement 22 days into medical therapy, on the 29th hospital day. Her valve was completely destroyed, but on culture was sterile. Antibiotics were continued for another 15 days; however, the patient had postoperative complications and died 4 weeks later.

### Case 3

A 53-year-old man underwent mitral valve replacement with a Starr Edwards metal valve. His postoperative course was complicated by acute renal failure, for which he required temporary hemodialysis by vascular catheter for 36 days. The maximum time a single line was in place was 13 days. Cultures of four vascular catheter tips after their removal grew both diphtheroids and coagulase-negative staphylococci, which were considered to be normal skin flora. During this time, a low-grade fever developed that resolved after a change of lines. Blood cultures remained sterile. Serial echocardiograms over the following 2 weeks, however, showed a worsening paraprosthetic leak. Subsequently, five sets of blood cultures grew *C. jeikeium* (Table 1). The patient completed 6 weeks of intravenous vancomycin and oral rifampicin for prosthetic valve endocarditis and was well on discharge. At follow-up, TOE studies showed no further increase in the paraprosthetic leak, and the patient had no clinical evidence of relapse of infection. He died 7 months later from noninfective causes.

## Discussion

Interest in the taxonomy of the nondiphtheriae corynebacteria has increased, with a resultant reclassification of earlier defined taxa and discovery of new pathogens in the group (*1*,*8*). One of the more commonly reported human infections with these organisms is infectious endocarditis. Since 1966, several case reports and reviews have described 191 cases of nondiphtheriae corynebacterial endocarditis on both prosthetic and native valves. In only six (3.1%) of these cases was association with an indwelling intravascular device specifically documented: two patients in association with chronic hemodialysis (*6*), one case attributed to an infected permanent pacemaker wire (*9*), two in connection with ventriculo-peritoneal shunts (*6*), and one in association with a ventriculo-atrial shunt (*10*). Of reports pertaining to hospital-acquired endocarditis in particular, in four case series totaling 80 cases, *Corynebacterium* species were not identified as causative agents (*7*,*11*–*13*). In another series, *Corynebacterium* species accounted for 1 (3.3%) of 30 cases (*14*), this case occurring postcardiac catheterization. A further series of 14 cases of hospital-acquired endocarditis describes 3 (21.4%) cases due to coryneform species but gives no species identification or source of infection (*15*).

We have described three cases of nondiphtheriae corynebacterial nosocomial endocarditis associated with IID, all occurring in a single hospital, accounting for one third of 10 cases occurring in an 18-month period. These cases include the first reported case of endocarditis caused by *C. amycolatum*. Of the remaining seven cases, two were due to methicillin-resistant *S. aureus* and five to methicillin-sensitive *S. aureus*.

Since Lehmann and Neumann proposed in 1896 that bacteria morphologically resembling the diphtheria bacillus be incorporated with it into the genus *Corynebacterium*, the classification of the coryneform bacteria has been drastically altered (*8*). This change has, in turn, increased the difficulty of identifying these organisms, as methods that reliably differentiate related species, such as mycolic acid chromatography, gas liquid chromatography, and molecular amplification techniques, cannot easily be used in the routine laboratory setting. During 1987 to 1995, 11 new *Corynebacterium* species were described (*1*). Commercial identification systems need to be updated to include those species relevant in human disease. *C. amycolatum* has only recently been included in the updated API Coryne database 2.0 (4), which, as illustrated by our case, may still lead to misidentification if used alone. That there have been no previous reports of *C. amycolatum* causing endocarditis may be due to its misidentification as other nonlipophilic fermentative corynebacteria species such as *C. xerosis* and *C. minutissimum* (*3*,*16*), both of which are associated with human disease.

Published material provides useful schema for differentiating *C. amycolatum*, *C. minutissimum*, and *C. striatum* by using colonial morphology, carbohydrate assimilation tests, and sensitivity to amoxycillin and the vibriostatic compound O/129, in conjunction with the API Coryne and API 20NE systems (*17*). Antibiotic sensitivity patterns may support identification, with *C. amycolatum* and *C. jeikeium* generally resistant to multiple antibiotics (*18*). In contrast, *C. striatum*, *C. minutissimum*, and *C. xerosis* generally are sensitive to a wide range of antibiotics.

Funke et al. (*1*) have published guidelines for identifying the coryneform bacteria, including simple phenotypic characteristics but also recommending more complex chemotaxonomic investigations and molecular genetic analysis if phenotypic characteristics do not differentiate between species. These guidelines are intended to facilitate the establishment of true disease associations of these organisms. In our experience, morphologic features combined with antibiogram profiles and the Coryne API allowed identification of two out of three corynebacteria (Table 2). As the colonial morphology and the Coryne API profile did not match in the isolate from Case 1, the organism was sent to our reference laboratory, where identification as *C. amycolatum* was confirmed by gas liquid chromatography (Microbial Identification System, Microbial ID, Newark, DE).

**Table 2:** Identification and antibiograms of the *Corynebacteria* species in three cases of endocarditis, London

Organism: gram-positive rod, nonmotile, catalase positive	Colonial morphology	Further identification	Antibiotic sensitivity pattern (Stokes plate)	
Sensitive	Resistant
*Corynebacterium amycolatum* Case 1	Dry, gray^a^	Coryne API GLC^b^	Rif, Teic, Vanc	Cip, Ery, F, Gent, Pen, Trim
*C. striatum* Case 2	Moist, white, smooth^a^	Coryne API	Cip, Ery, F, Gent, Rif, Trim, Teic, Vanc	Pen
*C. jeikeiun* Case 3	Gray, nonhemolytic^a^ (aerobic growth)	Coryne API	Rif, Teic, Vanc	Cip, Ery, F, Gent, Pen, Trim

^a^Horse blood agar at 37°C.

^b^API = analytical profile index; GLC = gas liquid chromatography; Cip = ciprofloxacin; Ery = erythromycin; F = fucidin; Gent = gentamicin; Pen = penicillin; Rif = rifampicin; Teic = teicoplanin; Trim = Trimethoprim; Vanc = vancomycin.

Treatment regimens described include penicillin alone, beta-lactam antibiotics or erythromycin plus gentamicin, and vancomycin. Our three patients all received dual therapy with vancomycin and rifampicin. This combination has not previously been described but was effective in two of the three patients (including the case of prosthetic valve endocarditis), who were both free from infection at least 5 months after cessation of therapy. In a review of 19 patients with prosthetic valve endocarditis due to diphtheroids (*19*), 4 (57.1%) of 7 patients treated with antibiotics alone were reported as cured at least 1 year posttreatment. Of 12 patients treated both medically and surgically, 7 (58.3%) were reported as having a microbiologic cure (*19*).

This study highlights the importance of the nondiphtheriae corynebacteria in severe human disease. Specifically, clinicians and microbiologists must be aware of the potential risk factors for nondiphtheriae corynebacterial endocarditis in the hospital setting and the danger of overlooking positive long-line tip cultures as normal skin flora or contaminants. Stringent identification of clinical isolates will be required to define the role of the nondiphtheriae corynebacteria in human disease.
